# Semen Quality Following Long-term Occupational Exposure to Formaldehyde in China

**DOI:** 10.1001/jamanetworkopen.2022.30359

**Published:** 2022-09-07

**Authors:** Mo-qi Lv, Hai-xu Wang, Yan-qi Yang, Rui-fang Sun, Pan Ge, Jian Zhang, Wen-bao Zhao, Shui-ping Han, Dang-xia Zhou

**Affiliations:** 1 Department of Pathology, School of Basic Medical Sciences, Medical School, Xi’an Jiaotong University, Xi’an, China; 2 Key Laboratory of Environment and Genes Related to Diseases, Ministry of Education, Xi’an, China; 3 Department of Obstetrics and Gynecology, the First Affiliated Hospital of the Fourth Military Medical University, Xi’an, China

## Abstract

**Importance:**

The potential effects of long-term occupational exposure to formaldehyde (FA) on human semen quality is not clear.

**Objective:**

To assess whether long-term occupational exposure to FA is associated with semen quality.

**Design, Setting, and Participants:**

This population-based cohort study was conducted from June 1 to June 30, 2021, in Xi’an, China. Participants were adults aged 23 to 40 years who had lived in the study area for 24 months or longer. Data analysis was performed from September 1 to October 1, 2021.

**Exposures:**

Long-term occupational exposure to FA was measured using a formaldehyde detector, and the FA exposure index (FEI) was calculated as follows: FEI = final concentration of FA (mg/m^3^) × work time during a workday (hour) × cumulative workdays (year).

**Main Outcomes and Measures:**

Semen samples were collected by masturbation after 3 to 7 days of abstinence and were then assessed by the computer-automated semen analysis system, Baso-Papanicolaou staining, and sperm-chromatin structure assay.

**Results:**

A total of 205 men (mean [SD] age, 29.49 [3.64] years), with 124 individuals in the FA exposure group (mean [SD] FEI, 73.72 [54.86]) and 81 age-matched controls, were included in the final analysis. Long-term personal occupational exposure to FA was significantly associated with poor semen quality. Specifically, a 1-unit increase in FEI was associated with a change of −0.99% (95% CI, −1.00% to −0.98%) in total sperm motility, −0.99% (95% CI, −0.99% to −0.97%) in progressive sperm motility, −0.05% (95% CI, −0.08% to −0.02%) in curvilinear velocity, −0.07% (95% CI, −0.10% to −0.04%) in straight line velocity, −0.07% (95% CI, −0.10% to −0.04%) in time-average velocity, −0.98% (95% CI, −0.99% to −0.93%) in normal sperm morphology, −0.24% (95% CI, −0.35% to −0.11%) in seminal neutral glucosidase, −0.61% (95% CI, −0.66% to −0.56%) in seminal plasma zinc, 0.52% (95% CI, 0.15% to 1.02%) in beat cross frequency, and 0.10% (95% CI, 0.06% to 0.14%) in the DNA fragmentation index. These associations remained significant after adjusting for confounding factors. Furthermore, subgroup analysis found that high levels of oxidative stress might promote the associations between FA exposure and semen quality.

**Conclusions and Relevance:**

This study found an association between long-term occupational exposure to FA and semen quality. This deterioration was dose and time dependent and might be induced by oxidative stress.

## Key Points

**Question:**

Is occupational exposure to formaldehyde (FA) in males associated with semen quality?

**Findings:**

In this cohort study of 205 men, long-term personal occupational exposure to FA was associated with lower semen quality. Additionally, this deterioration was dose and time dependent and might be induced by oxidative stress.

**Meaning:**

These findings may help researchers, physicians, and policy makers evaluate the hazardous effects of FA more completely and design targeted strategies for primary prevention of male infertility.

## Teaser

This cohort study assesses whether long-term occupational exposure to formaldehyde is associated with semen quality.

## Introduction

Infertility is a nonnegligible public health issue in male reproduction that is garnering increasing attention globally. Approximately 14% of couples in some high-resource countries and 25% of couples in some low-resource countries experience infertility.^1,2^ Male factors, mainly poor semen quality, account for nearly half of the infertility cases.^3^ A conspicuous decline in semen quality around the world^4,5,6,7^ confirmed this male reproductive crisis and triggered studies into the reason for decreases in semen quality. The remarkable changes in semen quality over a relatively short period is likely to be related to environmental factors.^8,9^

Formaldehyde (FA), a ubiquitous environmental contaminant, has been reported to be associated with various diseases, such as neurological, dermal, and respiratory disorders as well as cancer.^10,11^ As for the association with semen quality, numerous animal studies have found that FA exposure contributes to damage on seminiferous tubules^12,13,14,15,16,17^ and declines in semen quality^18,19,20^ in rats and mice. Unfortunately, human studies remained limited, and findings have been inconsistent. Ward et al^21^ reported that FA exposure showed no association with sperm count and morphology in Finnish males. However, this study was retrospective research relying on self-reported data and could be confounded by recall and selection biases. On the contrary, our previous study^22^ found that FA exposure was associated with decreased sperm motility in Chinese males. However, the uncomprehensive semen parameters in this previous study had limited capability to fully reflect the associations between FA exposure and semen quality.

Considering severe FA exposure^22,23,24,25^ and accelerating decline in semen quality in China,^7^ more studies are needed to explore whether occupational exposure to FA in males is associated with poor semen quality. Therefore, our goals were (1) to assess the association between long-term occupational exposure to FA and poor semen quality using the most comprehensive semen parameters including 20 parameters (5 conventional sperm parameters, 9 kinematic sperm parameters, sperm morphology, DNA fragmentation index [DFI], and 4 biochemistry parameters in seminal plasma) and (2) to explore the potential mechanism of associations between FA exposure and poor semen quality.

## Methods

### Study Participants

This population-based cohort study was conducted in the Shaanxi province of northwestern China from June 1 to June 30, 2021. The aim was to evaluate the associations of long-term occupational exposure to FA with semen quality. The study was organized by Xi’an Jiaotong Unversity. This work followed the Strengthening the Reporting of Observational Studies in Epidemiology (STROBE) reporting guideline and received approval for research ethics from the Medical Ethics Committee of Xi’an Jiaotong University. The study was conducted according to the Declaration of Helsinki.^26^ Volunteers were recruited from wood material industries in Xi’an. Participants were informed of the purpose of the present study and signed informed consent forms.

Inclusion criteria were as follows: (1) Chinese Han ethnicity; (2) aged 23 to 40 years; (3) worked in the FA exposure environment for at least 24 months and whose lifestyles did not change for at least 6 months prior to semen collection. To reduce the influence of confounding factors, exclusion criteria were carried out as follows: (1) living in newly built or decorated rooms; (2) having genital abnormalities or other chronic diseases including pelvic or spinal injuries, cytogenetic aberrations, testicular dysgenesis syndrome, sexually transmitted infections, chronic hepatitis B, hypertension, diabetes, and others; and (3) refusing to finish questionnaires and physical examination or to provide semen samples.

Reference group volunteers were recruited from the age-matched male Han population who had lived in same place and had no exposure experience to FA or other reproductive toxicants. Reference group volunteers were screened in accordance with the exclusion criteria.

### Questionnaire and Physical Examination

Questionnaires were designed based on our previous study^27^ and were completed by all participants, containing basic information (age, nationality, and income), lifestyle (smoking status, alcohol consumption), working information (workplace, work time during a workday, and cumulative workdays), occupational history, previous diseases, and abstinence duration. Meanwhile, a physical examination was conducted for every participant, including body mass index (BMI; calculated as weight in kilograms divided by height in meters squared) and genital examination.

### Exposure Assessment

According to the methods used in our previous study,^22^ the FA exposure index (FEI) was measured for every participant. The concentration of FA was measured for each participant using a formaldehyde detector. To reduce possible risks, we conducted the measurements at 3 different times during a workday (9:00 am, 12:00 pm, and 3:00 pm), and the mean value of the 3 measurements was used as the final concentration of FA. Then, combined with the information collected from the questionnaires, FEI was calculated as follows: FEI = the final concentration of FA (mg/m^3^) × work time during a workday (hour) × cumulative workdays (year).

### Semen Collection

Within 2 weeks after the FA exposure measurements, semen samples were collected by masturbation after 3 to 7 days of abstinence. All samples were incubated at 37 °C and analyzed within 60 minutes after collection.

### Determination of Semen Quality

#### Conventional Sperm Parameters

Semen analyses were conducted according to the World Health Organization (WHO) guidelines.^28^ A total of 5 conventional sperm parameters, including semen volume (SV), sperm concentration (SC), total sperm count (TSC), total sperm motility (TSM), and progressive sperm motility (PSM), were assessed by a well-trained technician using the computer automated semen analysis system (CASA [WLJY-9000]) according to the manufacturer’s guidelines. The detection method was described in our previous study.^22^ At least 200 sperm were detected from each sample, and the total sampling time for each sample lasted about 5 minutes. Among the 5 conventional sperm parameters, SV, SC, and TSC represented the sperm count, while TSM and PSM represented sperm motility.

#### Kinematic Sperm Parameters

Similar to the analysis of conventional sperm parameters, a total of 9 kinematic sperm parameters, including curvilinear velocity (VCL), straight line velocity (VSL), time-average velocity (VAP), mean angular displacement (MAD), linearity, straightness, wobble, amplitude of lateral head displacement (ALH), and beat cross frequency (BCF), were also estimated by the same CASA system according to the manufacturer’s guidelines.^22^ At least 200 sperm were detected from each sample and the total sampling time for each sample, lasted about 5 minutes. The 9 kinematic sperm parameters represented sperm motility.

#### Sperm Morphology

Sperm morphology analysis was performed using smears. After fixing semen in methanol, a Baso-Papanicolaou staining kit was used for staining the slides. According to the WHO guidelines,^28^ at least 200 sperm were examined from each sample with an optical microscope (Olympus) at ×400 magnification. The result of sperm morphology was calculated by the percentage of normal spermatozoa and was expressed as normal sperm morphology (NSM). The evaluations were performed by 2 independent researchers. Each researcher assessed the same smear independently, and the final NSM was the mean value of the 2 researchers. Any discrepancies greater than 10% were reexamined by a third researcher to reach a final consensus.

#### DFI

DFI evaluation was conducted using the sperm chromatin structure assay.^29^ Acridine orange was used to stain the sperm possessing unstable chromatin, and flow cytometers were used to collect fluorescence signals. A minimum of 10 000 events were recorded for each sample.

#### Biochemistry Parameters in Seminal Plasma

Seminal plasma was collected from each participant. The 4 biochemistry parameters, including acid phosphatase (ACP), neutral glucosidase (NG), fructose, and zinc were determined using commercial kits (Huakang) according to the manufacturer manual.

### Detection of Antioxidant Defenses in Semen

The antioxidant defenses in semen, including superoxide dismutase (SOD), malondialdehyde (MDA), and glutathione peroxidase (GSHPx), were determined using commercial kits (JianCheng) according to the manufacturer manual.

### Statistical Analysis

First, mean (SD) or median (IQR) for continuous variables and proportions for categorical variables were used to delineate demographic characteristics and semen quality. Second, the quantile-quartile plots were used to verify the normality of data, and any nonnormally distributed data was transformed into normally distributed data by the logarithmic transformation. Third, *t* and χ^2^ tests were used to explore the differences in all the characteristics aforementioned between the FA exposure group and control group. Fourth, a linear regression model was used to assess the associations between FEI and semen quality, controlling for confounding factors including age (years), BMI, income (yuan), smoking status, and alcohol consumption. Regression coefficients and 95% CIs were back-transformed and expressed as percentage change in each semen quality parameter for a 1-unit increase in FEI. Fifth, to investigate whether oxidative stress was a potential factor modifying the associations between FEI and semen quality, subgroup analyses were performed. The median level of SOD was identified and used as an empirical cutoff to dichotomize individuals into 2 groups: high and low levels of SOD. Linear regression models in low and high levels of SOD were conducted independently. Similarly, further subgroup analyses based on MDA and GSHPx were also conducted. Sixth, considering that smoking and drinking are known risk factors for poor sperm motility,^30,31,32^ a sensitivity analysis was performed by including and excluding them as confounding factors in linear regression models. In addition, we also conducted further analyses stratified by age and BMI to explore the potential effect modifiers.

All statistical analyses were performed in SPSS statistical software version 18.0 (IBM Corp). All tests were 2-sided, and *P* < .05 was considered statistically significant.

## Results

### Participant Characteristics

As shown in Table 1 and eFigure 1 in the Supplement, a total of 205 individuals were included in the final analysis, with a mean (SD) age of 29.49 (3.64) years, a mean (SD) income of 2463.42 (649.66) yuan ($364.90 [$96.23]), and a mean (SD) BMI of 24.01 (1.61); 174 men (84.88%) smoked, whereas 125 (60.98%) reported alcohol consumption. Among the 205 individuals, including 124 men with FA exposure and 81 in the control group, no differences between exposure group and control group were seen in age, income, BMI, smoking status, or alcohol consumption (eFigure 2 in the Supplement).

**Table 1.** Main Characteristics and Semen Parameters of the Study Participants

Characteristic	Participants, No. (%) (N = 205)
Age, mean (SD), y	29.49 (3.64)
BMI, mean (SD)	24.01 (1.61)
Income, yuan/working period, mean (SD)^a^	2463.42 (649.66)
Smoking status	
Nonsmoker	31 (15.12)
Smoker	174 (84.88)
Alcohol consumption	
Nondrinker	80 (39.02)
Drinker	125 (60.98)
Conventional sperm parameters, median (IQR)	
SV, mL	2.5 (2.00-3.60)
SC, million/mL	54.07 (34.49-74.73)
TSC, million	128.79 (79.20-206.33)
TSM, %	51.01 (40.53-66.54)
PSM, %	40.13 (29.92-54.28)
Kinematic sperm parameters, mean (SD), μm/s	
VCL	49.74 (10.14)
VSL	33.16 (7.86)
VAP	36.13 (7.96)
MAD, mean (SD), °	53.86 (8.05)
Linearity, mean (SD)	65.21 (8.23)
Straightness, mean (SD)	88.12 (4.07)
Wobble, mean (SD)	72.24 (6.74)
ALH, mean (SD), μm	3.44 (1.12)
BCF, mean (SD)	5.09 (1.03)
NSM, median (IQR), %	10 (7.00-15.00)
DFI, mean (SD), %	18.85 (7.99)
Biochemistry parameters in seminal plasma, mean (SD)	
ACP, U/ejaculate	71.05 (21.08)
NG, mU/ejaculate	7.4 (1.8)
Fructose, μmol/ejaculate	4.96 (0.86)
Zinc, μmol/ejaculate	4.76 (1.62)

Abbreviations: ACP, acid phosphatase; ALH, amplitude of lateral head displacement; BCF, beat cross frequency; BMI, body mass index (calculated as weight in kilograms divided by height in meters squared); DFI, DNA fragmentation index; MAD, mean angular displacement; NG, neutral glucosidase; NSM, normal sperm morphology; PSM, progressive sperm motility; SC, sperm concentration; SV, semen volume; TSC, total sperm count; TSM, total sperm motility; VAP, average path velocity; VCL, curvilinear velocity; VSL, straight line velocity.

^a^ To convert yuan to US dollars, multiply by 0.15.

### FA Exposure Level

Among 124 individuals in the FA exposure group, the mean (SD) concentration of FA was 1.49 (0.61) mg/m^3^, and the mean (SD) FEI was 73.72 (54.86). For the 81 men in the control group, FA occupational exposure could be ignored because of the low concentration of FA in the air (0-0.02 mg/m^3^).

### Semen Quality With and Without Occupational Exposure to FA

#### Conventional Sperm Parameters

**Figure 1.**
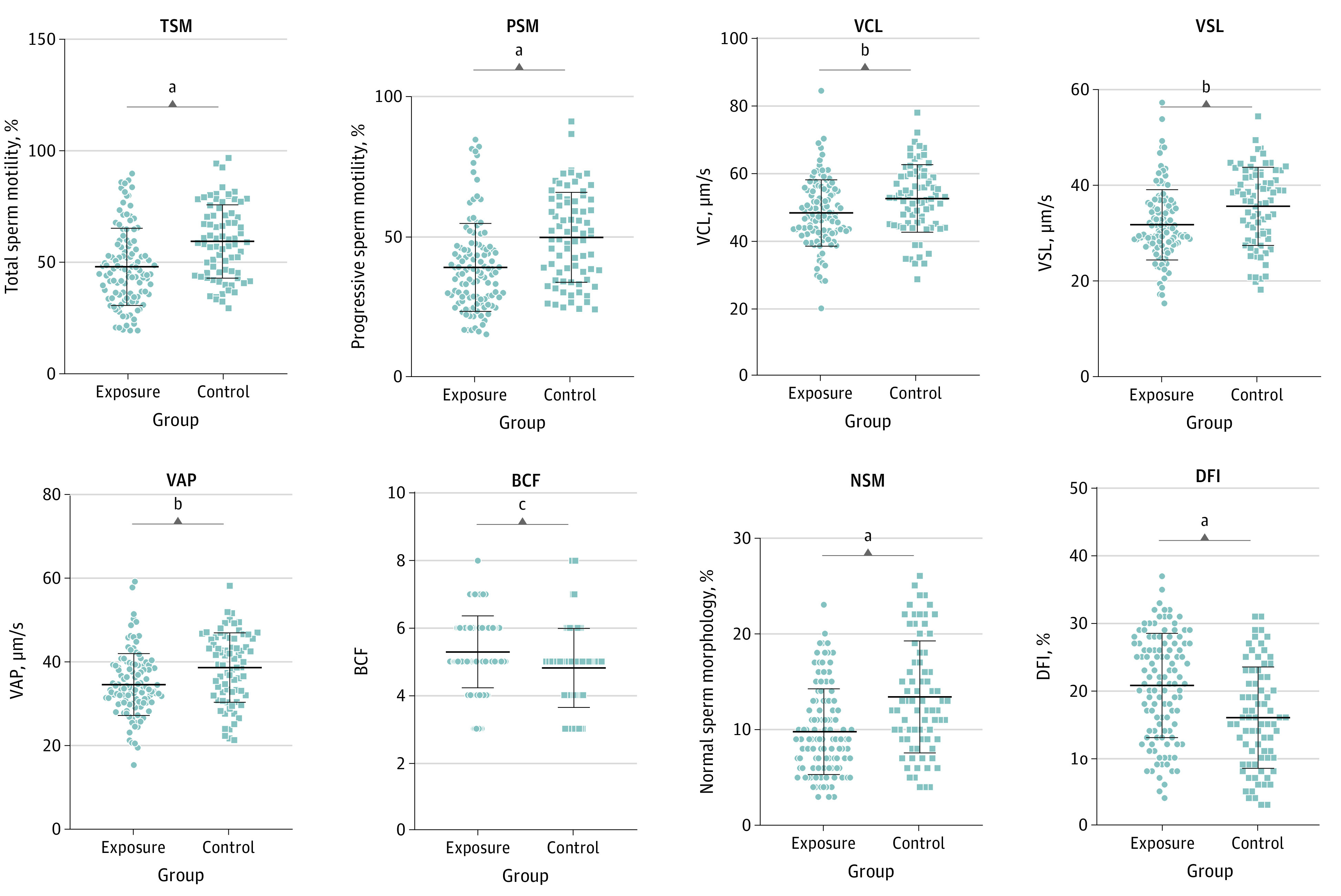
Semen Quality With and Without Occupational Formaldehyde Exposure The center line is the mean (for curvilinear velocity [VCL], straight line velocity [VSL], average path velocity [VAP], beat cross frequency [BCF], and DNA fragmentation index [DFI]) or median (for total sperm motility [TSM], progressive sperm motility [PSM], and normal sperm morphology [NSM]), with outer lines indicating SD (for VCL, VSL, VAP, BCF, and DFI) or IQR (for TSM, PSM, and NSM). Each marker represents an individual’s data. ^a^*P* < .001. ^b^*P* < .005. ^c^*P* < .01.

As shown in Figure 1, men in the FA exposure group, compared with the control group, had lower median (IQR) TSM (46.54% [35.15%-58.84%] vs 58.33% [45.21%-73.72%]; *P* < .001) and PSM (37.12% [27.75%-46.08%] vs 50.11% [30.66%-64.42%]; *P* < .001). On the other hand, the differences in SV (2.60 [1.75-3.60] mL vs 2.50 [2.10-3.50] mL; *P* = .45), SC (54.25 [33.26-73.5] million/mL vs 53.94 [34.08-77.83] million/mL; *P* =.65), and TSC (125.20 [73.69-192.30] million vs 129.11 [76.62-221.29] million; *P* = .42) were not statistically significant (eFigure 5 in the Supplement).

#### Kinematic Sperm Parameters

Comparing men with vs without FA exposure, mean (SD) VCL (48.02 [9.89] μm/s vs 53.19 [9.77] μm/s; *P* = .004), VSL (31.62 [7.33] μm/s vs 34.99 [7.85] μm/s; *P* = .001), and VAP (34.52 [7.38] μm/s vs 39.22 [9.60] μm/s; *P* = .001) were lower; however, BCF (5.25 [0.97] vs 4.85 [1.09]; *P* = .009) was higher in the FA exposure group vs the control group (Figure 1). MAD, linearity, straightness, wobble, and ALH showed no changes between the exposure and control groups (eFigure 5 in the Supplement).

#### Sperm Morphology and DFI

The median (IQR) value of normal sperm morphology (NSM) in the FA exposure group and control group was 9.79% (6.00%-13.00%) and 13.41% (9.00%-17.75%), respectively, indicating that lower NSM was associated with exposure to FA (*P* < .001) (Figure 1). On the contrary, the mean value of DFI in FA exposure group and control group was 20.76% (7.75%) and 15.97% (7.53%), respectively, suggesting that FA is associated with increased DFI (*P* < .001) (Figure 1).

#### Biochemistry Parameters in Seminal Plasma

In the FA exposure group, zinc was lower than in the control group (eFigure 5 in the Supplement). ACP, NG, and fructose did not indicate any significant differences (eFigure 5 in the Supplement).

### Association Between FEI and Semen Quality

#### Association Between FEI and Conventional Sperm Parameters

**Figure 2.**
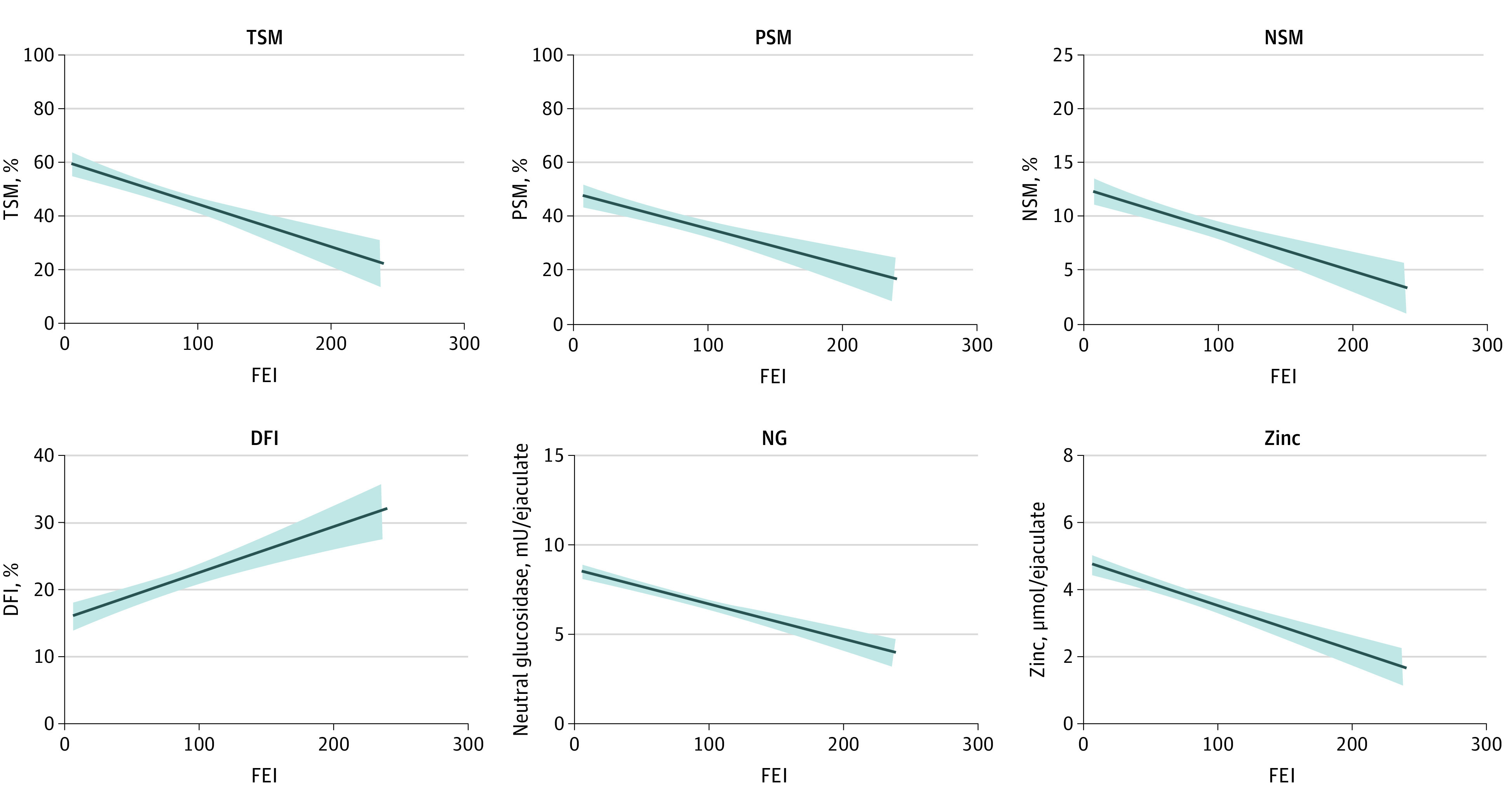
Associations of Formaldehyde Exposure Index (FEI) With Semen Quality Solid curves indicate the effect estimate; shaded areas, 95% CIs; DFI, DNA fragmentation index; NG, neutral glucosidase; NSM, normal sperm morphology; PSM, progressive sperm motility; and TSM, total sperm motility.

Figure 2 and Table 2 summarize the results of linear regression. The FEI was negatively associated with TSM and PSM, meaning that a change of −0.99% (95% CI, −1.00% to −0.98%; *P* <.001) in TSM and −0.99% (95% CI, −0.99% to −0.97%; *P* <.001) in PSM were associated with a 1-unit increase in FEI. SV, SC, and TSC had no statistically significant association with FEI (Table 2). In addition, after adjusting for confounding factors, including age, smoking, alcohol consumption, body mass index, and income, the associations between FEI and conventional sperm parameters showed the same trends (Table 2; eFigure 3 in the Supplement).

**Table 2.** Associations of FEI With Semen Quality and Biochemistry Parameters of Seminal Plasma Before and After Adjusting for Confounding Factors^a^

Parameter	All			
	Crude		Adjusted	
	Effect estimate, % (95% CI)^b^	*P* value	Effect estimate, % (95% CI)^b^	*P* value
SV	−0.56 (−0.90 to 0.89)	.26	−0.45 (−0.87 to 1.42)	.42
SC	−0.11 (−0.69 to 1.58)	.81	−0.01 (−0.66 to 1.91)	.98
TSC	−0.34 (−0.74 to 0.65)	.36	−0.22 (−0.69 to 1.00)	.60
TSM	−0.99 (−1.00 to −0.98)	<.001^c^	−0.99 (−1.00 to −0.98)	<.001^c^
PSM	−0.99 (−0.99 to −0.97)	<.001^c^	−0.99 (−0.99 to −0.98)	<.001^c^
VCL	−0.05 (−0.08 to −0.02)	<.001^c^	−0.05 (−0.08 to −0.02)	<.001^c^
VSL	−0.07 (−0.10 to −0.04)	<.001^c^	−0.08 (−0.11 to −0.04)	<.001^c^
VAP	−0.07 (−0.10 to −0.04)	<.001^c^	−0.08 (−0.11 to −0.04)	<.001^c^
MAD	0.01 (−0.02 to 0.05)	.47	0.02 (−0.01 to 0.05)	.27
Linearity	−0.02 (−0.06 to 0.00)	.09	−0.03 (−0.07 to 0.00)	.05
Straightness	−0.06 (−0.12 to 0.00)	.09	−0.07 (−0.13 to 0.00)	.05
Wobble	−0.03 (−0.07 to 0.00)	.10	0.00 (−0.08 to 0.00)	.05
ALH	−0.06 (−0.28 to 0.22)	.62	−0.06 (−0.28 to 0.21)	.60
BCF	0.52 (0.15 to 1.02)	.003^c^	0.57 (0.18 to 1.08)	.002^c^
NSM	−0.98 (−0.99 to −0.93)	<.001^c^	−0.98 (−0.99 to −0.93)	<.001^c^
DFI	0.10 (0.06 to 0.14)	<.001^c^	0.10 (0.06 to 0.14)	<.001^c^
ACP	0.00 (−0.01 to 0.01)	.71	0.00 (−0.01 to 0.01)	.72
NG	−0.24 (−0.35 to −0.11)	.001^c^	−0.24 (−0.35 to −0.10)	.001^c^
Fructose	0.19 (−0.15 to 0.69)	.30	0.27 (−0.09 to 0.79)	.16
Zinc	−0.61 (−0.66 to −0.56)	<.001^c^	−0.61 (−0.65 to −0.56)	<.001^c^

Abbreviations: ACP, acid phosphatase; ALH, amplitude of lateral head displacement; BCF, beat cross frequency; DFI, DNA fragmentation index; FEI, formaldehyde exposure index; MAD, mean angular displacement; NG, neutral glucosidase; NSM, normal sperm morphology; PSM, progressive sperm motility; SC, sperm concentration; SV, semen volume; TSC, total sperm count; TSM, total sperm motility; VAP, average path velocity; VCL, curvilinear velocity; VSL, straight line velocity.

^a^ Confounding factors used in the adjusted model contained age, body mass index, income, smoking status, and alcohol consumption.

^b^ Effect estimates represent percentage changes in association with a 1-unit increase in FEI.

^c^ *P* < .05.

#### Association Between FEI and Kinematic Sperm Parameters

As shown in eFigure 6 in the Supplement and Table 2, FEI was significantly inversely associated with VCL, VSL, and VAP and positively associated with BCF. Specifically, a 1-unit increase in FEI was associated with a change of −0.05% (95% CI, −0.08% to −0.02%; *P* < .001) in VCL, −0.07% (95% CI, −0.10% to −0.04%; *P* < .001) in VSL, −0.07% (95% CI, −0.10% to −0.04%; *P* < .001) in VAP, and 0.52% (95% CI, 0.15% to 1.02%; *P* = .003) in BCF. However, MAD, linearity, straightness, wobble, and ALH had no significant association with FEI (Table 2). Additionally, the associations with the same trends between FEI and kinematic sperm parameters were observed after adjusting for confounding factors (Table 2; eFigure 3 in the Supplement).

#### Association Between FEI and Sperm Morphology and DFI

We found a negative association between FEI and NSM and a positive association between FEI and DFI (Figure 2 and Table 2). Specifically, a 1-unit increase in FEI was associated with a change of −0.98% (95% CI, −0.99% to −0.93%; *P* < .001) in NSM and an increase of 0.10% (95% CI, 0.06% to 0.14%; *P* < .001) in DFI. After adjusting for confounding factors, the 2 associations remained significant (Table 2; eFigure 3 in the Supplement).

#### Association Between FEI and Biochemistry Parameters in Seminal Plasma

As shown in Figure 2 and Table 2, NG and zinc were negatively associated with FEI. Specifically, a 1-unit increase in FEI was associated with a change of −0.24% (95% CI, −0.35% to −0.11%; *P* = .001) in NG and −0.61% (95% CI, −0.66% to −0.56%; *P* < .001) in zinc. However, ACP and fructose were not associated with FEI (Table 2). After adjusting for confounding factors, similar associations with narrower 95% CIs were observed for biochemistry parameters (Table 2; eFigure 3 in the Supplement).

### Subgroup Analyses of Semen Quality Based on Different Levels of Antioxidant Defenses

We further divided the men with FA exposures into groups based on antioxidant defenses. They were divided into low and high categories based on the median level of SOD (134.87 U/ml), MDA (6.49 nmol/ml), and GSHPx (54.44 U/ml).

#### Subgroup Analyses of Semen Quality Based on Different Levels of SOD

**Figure 3.**
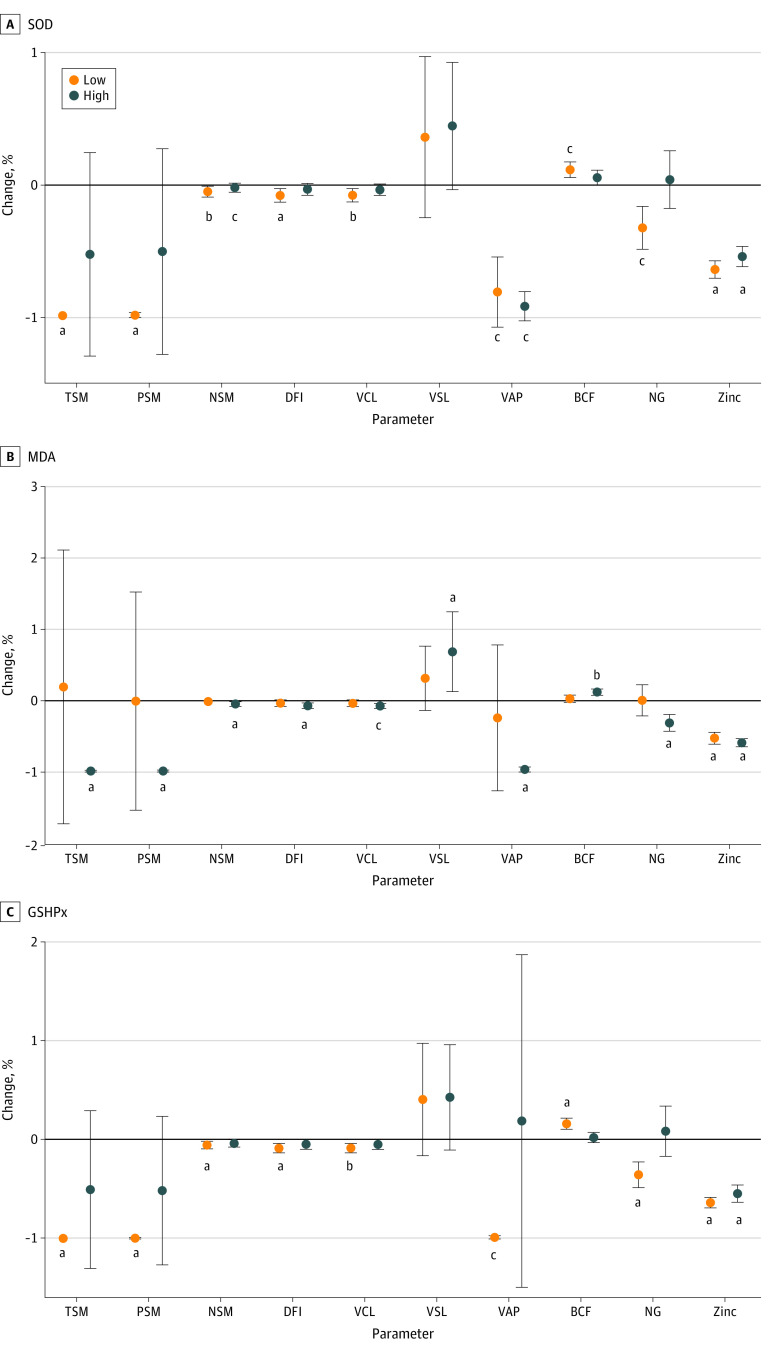
Percentage Changes in Semen Quality Associated With a 1-Unit Increase in Formaldehyde Exposure Index in Different Level of Antioxidant Defenses Dots indicate the percentage changes associated with a 1-unit increase in the formaldehyde exposure index; error bars, 95% CIs; BCF, beat cross frequency; DFI, DNA fragmentation index; GSHPx, glutathione peroxidase; MDA, malondialdehyde; NSM, normal sperm morphology; NG, neutral glucosidase; PSM, progressive sperm motility; SOD, superoxide dismutase; TSM, total sperm motility; VAP, average path velocity; VCL, curvilinear velocity; and VSL, straight line velocity. ^a^*P* < .001. ^b^*P* < .01. ^c^*P* < .005.

In the subgroup analysis of SOD levels, the associations between semen quality and FEI remained significant in the group with low levels of SOD but not in the group with high levels (Figure 3; eTable 1 in the Supplement). Specifically, the associations between the FEI and some measures of semen quality were significant in the subgroup with low SOD (TSM: percentage change, −0.99%; 95% CI, −1.00% to −0.97%; *P* < .001; PSM: percentage change, −0.99%; 95% CI, −1.00% to −0.96%; *P* < .001; VCL: percentage change, −0.05%; 95% CI, −0.09% to −0.01%; *P* = .009; VSL: percentage change, −0.08%; 95% CI, −0.13% to −0.03; *P* = .003; VAP: percentage change, −0.08%; 95% CI, −0.13% to −0.03%; *P* = .002; NG: percentage change, −0.34%; 95% CI, −0.48% to −0.16%; *P* = .001; DFI: percentage change, 0.10%; 95% CI, 0.05% to 0.16%; *P* < .001). NSM and zinc had statistically significant negative associations in both groups (NSM, low level of SOD: percentage change, −0.93%; 95% CI, −0.99% to −0.50%; *P* = .009; NSM, high level of SOD: percentage change, −0.97%; 95% CI, −0.99% to −0.79%; *P* = .001; zinc, low level of SOD: percentage change, −0.64%; 95% CI, −0.70% to −0.57%; *P* < .001; zinc, high level of SOD: percentage change, −0.54%; 95% CI, −0.62% to −0.46%; *P* < .001). BCF had no association in either group for SOD (eFigure 4 in the Supplement).

#### Subgroup Analyses of Semen Quality Based on Different Levels of MDA

Contrary to the subgroup analysis of SOD, nearly all the parameters mentioned previously were associated with FEI in the group with higher levels of MDA but not in the group with lower levels (Figure 3; eTable 2 in the Supplement). In particular, the negative associations in TSM (percentage change, −0.99%; 95% CI, −0.99% to −0.97%; *P* < .001), PSM (percentage change, −0.99%; 95% CI, −0.99% to −0.97%; *P* < .001), VCL (percentage change, −0.05%; 95% CI, −0.08% to −0.02%; *P* = .002), VSL (percentage change, −0.07%; 95% CI, −0.11% to −0.03%; *P* < .001), VAP (percentage change, −0.08%; 95% CI, −0.11% to −0.04%; *P* < .001), NSM (percentage change, −0.98%; 95% CI, −0.99% to −0.92%; *P* < .001), and NG (percentage change, −0.32%; 95% CI, −0.42% to −0.19%; *P* < .001) as well as the positive associations with DFI (percentage change, 0.11%; 95% CI, 0.07% to 0.15%; *P* < .001) and BCF (percentage change, 0.61%; 95% CI, 0.15% to 1.26%; *P* = .006) remained statistically significant in the group with higher levels but not in the group with lower levels. Zinc showed converse associations in both low and high groups of MDA (low level of MDA: percentage change, −0.53%; 95% CI, −0.61% to −0.44%; *P* < .001; high level of MDA: percentage change, −0.59%; 95% CI, −0.64% to −0.53%; *P* < .001) (eFigure 4 in the Supplement).

#### Subgroup Analyses of Semen Quality Based on Different Levels of GSHPx

Similar to the subgroup analysis of SOD, most parameters mentioned previously had significant associations with FEI in the group with low levels of GSHPx but not in the group with high levels (Figure 3; eTable 3 in the Supplement). The negative associations with TSM (percentage change, −0.99%; 95% CI, −0.99% to −0.98%; *P* < .001), PSM (percentage change, −0.99%; 95% CI, −0.99% to −0.98%; *P* < .001), VCL (percentage change, −0.05%; 95% CI, −0.09% to −0.01%; *P* = .007), VSL (percentage change, −0.08%; 95% CI, −0.13% to −0.03%; *P* = .001), VAP (percentage change, −0.08%; 95% CI, −0.13% to −0.03%; *P* = .001), NSM (percentage change, −0.99%; 95% CI, −0.99% to −0.96%; *P* < .001), and NG (percentage change, −0.36%; 95% CI, −0.47% to −0.22%; *P* < .001) as well as the positive association with DFI (percentage change, 0.16%; 95% CI, 0.10% to 0.21%; *P* < .001) were statistically significant in the group with low levels of GSHPx but not in the group with high levels. Zinc showed statistically negative associations in both groups; however, BCF was not associated with FEI in either group (low level of GSHPx: percentage change, −0.63%; 95% CI, −0.68% to −0.58%; *P* < .001; high level of GSHPx: percentage change, −0.55%; 95% CI, −0.62% to −0.45%; *P* < .001) (eFigure 4 in the Supplement). The results in further subgroup analyses adjusting for confounding factors were almost unchanged (eFigure 4, eTable 1, eTable 2, and eTable 3 in the Supplement).

### Sensitivity Analyses and Stratified Analyses

Sensitivity analyses showed that results of the unadjusted analysis (smoking and alcohol consumption) were generally similar to those of adjusted analysis, although effect estimates became relatively larger (eTable 4 in the Supplement). Stratified analyses indicated stronger associations between FEI and BCF in men younger than 30 years or with a BMI lower than 24. However, these differences in estimated effects were generally nonsignificant (ie, had overlapping 95% CIs). Associations between FEI and other semen quality parameters in different stratified groups were generally similar to those in overall groups (eTable 5 in the Supplement).

## Discussion

The present study assessed the associations between FA exposure and semen quality using 20 semen parameters, which we believe to be the most comprehensive evaluation system. Our results indicated that a long-term occupational exposure to FA was associated with a serious deterioration in sperm motility, morphology, and DNA integrity. Specifically, an increase in FEI was associated with decreases in TSM, PSM, VCL, VSL, VAP, NSM, and seminal plasma zinc as well as increases in BCF and DFI before and after adjusting for confounding factors, indicating that the associations were dose and time dependent. Furthermore, oxidative stress in semen seemed to enhance the association of FA exposure with semen quality, suggesting that the negative associations of occupational exposure to FA with semen quality might be induced by oxidative stress. To our knowledge, this is the first population-based cohort study to explore the potential mechanism of the associations between FA exposure and semen quality.

Studies on the associations between FA exposure and semen quality have been limited, and findings have remained inconsistent. In Finland, FA exposure was not associated with changes in sperm count and morphology.^21^ However, in China, our previous study^22^ found that FA exposure in males was associated with decreased sperm motility. The differences might result from racial and ethnic differences, representing various gene phenotypes and lifestyles that may affect semen quality.^33^ The present findings were coincident with our previous results^22^; the participants we recruited in this study had similar ethnicity and residence and came from the similar population. In addition, our previous study evaluated the semen quality with 5 conventional and kinematic parameters; however, these parameters were insufficient given that sperm morphology and sperm DNA integrity, essential assessors of semen quality and fertilization potential, were not added in the study. Our present study made up for this deficiency and found that sperm morphology and sperm DNA integrity were also associated with occupational FA exposure.

The present findings, ie, long-term exposure to FA was associated with significant reductions in sperm motility, morphology, and DFI, were in agreement with animal studies.^34,35,36,37,38^ Significantly, nearly all the animal studies reported that FA exposure could decrease sperm number^34,35,36,37,38^; however, similar declines were not observed in our study nor in the study by Wang et al.^22^ The differences might result from different doses and time of exposure. Animals were exposed to a relative high dose (10 mg/m^3^) for a short period (<35 d),^36^ while humans were exposed to a relatively low dose (0.22-5.43 mg/m^3^) for a longer period (>24 months). Moreover, a histological study of testis in animals revealed destruction of germinal epithelium, spermatogenic arrest, vacuolization of the tubules, and degeneration of Leydig cells.^34,36,37^ The disorder in spermatogenesis might be the source of weak quality in sperm.

Our study further explored the possible mechanism of the association of FA exposure with poor semen quality. Previous studies indicated that exposure to FA might increase the level of oxidative stress, resulting in the tissue damage and inflammation.^39^ In addition, some environmental pollutants and viruses induce poor semen quality through increasing levels of oxidative stress.^40,41,42^ Additionally, antioxidant treatment has been shown to have a positive effect on semen quality.^43,44^ Moreover, several antioxidant or oxidant compounds were found to alleviate oxidative damage in sperm caused by FA exposure.^34,37^ SOD and GSHPx are antioxidant enzymes protecting cells against oxidative damage, and MDA is a oxidation parameter measuring lipid peroxidation.^45^ It has been reported that FA exposure significantly decreases the levels of SOD and GSHPx, while it increased the level of MDA in the testicular tissue, suggesting that FA is associated with testicular oxidative stress and impaired testicular tissue in rodents.^46^ This result is consistent with our findings. Our study, to our knowledge, was the first population-based cohort study to investigate the association of oxidative stress with the association between FA exposure and semen quality. We found that in individuals with low SOD, high MDA, or low GSHPx, which indicate high levels of oxidative stress, FEI was negatively associated with semen quality; otherwise, in individuals with low levels of oxidative stress, no association was observed. This finding suggests that associations between FA exposure and poor semen quality might be induced by oxidative stress. Further studies are expected to add an intervention group receiving antioxidants.

### Strengths and Limitations

The present study had several novel strengths. First, our research formulated strict inclusion and exclusion criteria to eliminate the selection bias coming from volunteers who had insufficient exposure time (<24 months); who lived in residences with high levels of indoor FA, such as a newly built or remodeled apartment; and who showed vast differences in educational levels and socioeconomic status as control group. These individuals were excluded given potential selection bias.^22^ Second, to our knowledge, our study used the most comprehensive set of 20 semen parameters, including 5 conventional sperm parameters (SV, SC, TSC, TSM, and PSM), 9 kinematic sperm parameters (VCL, VSL, VAP, MAD, linearity, straightness, wobble, ALH, and BCF), sperm morphology, DNA fragmentation index, and 4 biochemistry parameters of seminal plasma (ACP, NG, fructose, and zinc), to examine the associations between long-term occupational FA exposure and male reproductive health. Third, the present study used precise exposure monitoring equipment and a scientific exposure method to obtain accurate exposure data for participants. In addition, exposure durations were added in our analysis to calculate FEI according to both exposure duration and concentration. This technique was more precise in reflecting the true occupational FA exposure level. Fourth, our study was the first population-based cohort study to explore the possible mechanism of inverse associations between FA exposure and semen quality, and our result verified that oxidative stress in semen could promote the negative effect of FA occupational exposure on semen quality, which had been reported in animal studies.^34,37^

Although strict and rigorous methods were used to reduce bias, our study also had an unavoidable limitation. Only the concentration of FA in the workplace was measured and analyzed in our study. However, the background level of each individual was not measured. We recruited exposure and control groups from the same living place to alleviate these potential sources of bias. The varying metabolic capacity of FA was another factor contributing to the different background levels. The same concentration of FA in air could have various impacts in different participants. More precise indicators, such as formic acid in urine, a formaldehyde metabolite, should be analyzed in further studies to understand the association between FA in body and semen quality. In addition, the present study was conducted in a single region with a limited sample size, which may contribute to selection bias. This limited population may affect the generalizability of the study results. Further nationally retrospective cohort studies with larger sample size are in needed.

## Conclusions

In this study, long-term occupational exposure to FA was negatively associated with semen quality, especially with sperm motility, morphology, and DNA integrity. In addition, our results indicated that the negative associations were dose and time dependent. Moreover, the associations of FA occupational exposure with poor semen quality might be induced by oxidative stress. The present study strengthened the molecular epidemiological understanding of FA exposure and semen quality and increased the objective evidence of the negative association of FA exposure with male reproduction.

## Notes

Supplement. **eTable 1.** Associations of FEI With Semen Quality and Biochemistry Parameters of Seminal Plasma in Different Level of SOD Before and After Adjusting for Confounding Factors **eTable 2.** Associations of FEI With Semen Quality and Biochemistry Parameters of Seminal Plasma in Different Level of MDA Before and After Adjusting for Confounding Factors **eTable 3.** Associations of FEI With Semen Quality and Biochemistry Parameters of Seminal Plasma in Different Level of GSHPx Before and After Adjusting for Confounding Factors **eTable 4.** Effect Estimates and 95% CIs of Semen Quality Parameters With a 1-Unit Increase in FEI With and Without Adjustment for Smoking and Alcohol Consumption **eTable 5.** Effect Estimates and 95% CIs of Semen Quality Parameters Associated With a 1-Unit Increase in FEI in Each Subgroup **eFigure 1.** Flowchart of Participant Recruitment **eFigure 2.** Distribution of Sociodemographic Characteristics With and Without FA Exposure **eFigure 3.** Percentage Changes in Semen Quality Associated With a 1-Unit Increase in FEI Before and After Adjusting for Confounding Factors **eFigure 4.** Heatmap of the Associations Between FEI and Semen Quality in Different Groups of Antioxidant Defenses Before and After Adjusting for Confounding Factors **eFigure 5.** Distribution of Semen Quality With and Without Occupational FA Exposure **eFigure 6.** Significant Associations of FEI With Kinematic Sperm Parameters
