# Error in Article Information

**DOI:** 10.1001/jamanetworkopen.2022.38925

**Published:** 2022-10-04

In the Original Investigation titled “Semen Quality Following Long-term Occupational Exposure to Formaldehyde in China,”^1^ published September 7, 2022, there was an error in the Article Information section. The Funding/Support section did not list grant 2022ZDLSF03-10 from the Key Research Plan of Shaanxi Province. This article has been corrected.^1^
