# Supplementary material for: Semen Quality Following Long-term Occupational Exposure to Formaldehyde in China
Source: JAMA Netw Open. 2022 Sep 7;5(9):e2230359. doi: 10.1001/jamanetworkopen.2022.30359 (PMC9453542; doi:10.1001/jamanetworkopen.2022.30359)
Supplement: Supplement. — eTable 1. Associations of FEI With Semen Quality and Biochemistry Parameters of Seminal Plasma in Different Level of SOD Before and After Adjusting for Confounding Factors eTable 2. Associations of FEI With Semen Quality and Biochemistry Parameters of Seminal Plasma in Different Level of MDA Before and After Adjusting for Confounding Factors eTable 3. Associations of FEI With Semen Quality and Biochemistry Parameters of Seminal Plasma in Different Level of GSHPx Before and After Adjusting for Confounding Factors eTable 4. Effect Estimates and 95% CIs of Semen Quality Parameters With a 1-Unit Increase in FEI With and Without Adjustment for Smoking and Alcohol Consumption eTable 5. Effect Estimates and 95% CIs of Semen Quality Parameters Associated With a 1-Unit Increase in FEI in Each Subgroup eFigure 1. Flowchart of Participant Recruitment eFigure 2. Distribution of Sociodemographic Characteristics With and Without FA Exposure eFigure 3. Percentage Changes in Semen Quality Associated With a 1-Unit Increase in FEI Before and After Adjusting for Confounding Factors eFigure 4. Heatmap of the Associations Between FEI and Semen Quality in Different Groups of Antioxidant Defenses Before and After Adjusting for Confounding Factors eFigure 5. Distribution of Semen Quality With and Without Occupational FA Exposure eFigure 6. Significant Associations of FEI With Kinematic Sperm Parameters [file jamanetwopen-e2230359-s001.pdf]

Supplemental Online Content

Lv MQ, Wang HX, Yang YQ, et al. Semen quality following long-term occupational exposure to formaldehyde in China. *JAMA Netw Open.* 2022;5(9):e2230359. doi:10.1001/jamanetworkopen.2022.30359

eTable 1. Associations of FEI With Semen Quality and Biochemistry Parameters of Seminal Plasma in Different Level of SOD Before and After Adjusting for Confounding Factors

eTable 2. Associations of FEI With Semen Quality and Biochemistry Parameters of Seminal Plasma in Different Level of MDA Before and After Adjusting for Confounding Factors

eTable 3. Associations of FEI With Semen Quality and Biochemistry Parameters of Seminal Plasma in Different Level of GSHPx Before and After Adjusting for Confounding Factors

eTable 4. Effect Estimates and 95% CIs of Semen Quality Parameters With a 1-Unit Increase in FEI With and Without Adjustment for Smoking and Alcohol Consumption

eTable 5. Effect Estimates and 95% CIs of Semen Quality Parameters Associated With a 1-Unit Increase in FEI in Each Subgroup

eFigure 1. Flowchart of Participant Recruitment

eFigure 2. Distribution of Sociodemographic Characteristics With and Without FA Exposure

eFigure 3. Percentage Changes in Semen Quality Associated With a 1-Unit Increase in FEI Before and After Adjusting for Confounding Factors

eFigure 4. Heatmap of the Associations Between FEI and Semen Quality in Different Groups of Antioxidant Defenses Before and After Adjusting for Confounding Factors

eFigure 5. Distribution of Semen Quality With and Without Occupational FA Exposure

eFigure 6. Significant Associations of FEI With Kinematic Sperm Parameters

This supplemental material has been provided by the authors to give readers additional information about their work.

© 2022 Lv M et al. *JAMA Network Open.*

eTable 1. Associations of FEI With Semen Quality and Biochemistry Parameters of Seminal Plasma in Different Level of SOD Before and After Adjusting for Confounding Factors[#]

	Low level of SOD								High level of SOD							
	Crude				Adjusted				Crude				Adjusted			
	Effect estimate[b](%)	95%CI lower	95%CI higher	*P* value	Effect estimate[b](%)	95%CI lower	95%CI higher	*P* value	Effect estimate[b](%)	95%CI lower	95%CI Higher	*P* value	Effect estimate[b](%)	95%CI lower	95%CI higher	*P* value
TSM	-0.99	-1.00	-0.97	<0.001[c]	-0.99	-1.00	-0.95	<0.001[c]	-0.94	-0.99	0.35	0.076	-0.97	-0.99	0.56	0.066
PSM	-0.99	-1.00	-0.96	<0.001[c]	-0.99	-0.99	-0.95	<0.001[c]	-0.91	-0.99	0.38	0.083	-0.96	-0.99	1.75	0.070
VCL	-0.05	-0.09	-0.01	0.009[c]	-0.05	-0.09	-0.01	0.015[c]	-0.02	-0.06	0.00	0.147	-0.02	-0.06	0.00	0.103
VSL	-0.08	-0.13	-0.03	0.003[c]	-0.08	-0.13	-0.03	0.002[c]	-0.03	-0.08	0.00	0.086	-0.04	-0.09	0.00	0.055
VAP	-0.08	-0.13	-0.03	0.002[c]	-0.08	-0.13	-0.03	0.002[c]	-0.04	-0.08	0.00	0.066	-0.05	-0.09	0.07	0.069
BCF	0.26	-0.20	1.00	0.313	0.38	-0.11	1.16	0.155	0.38	-0.01	0.94	0.058	0.44	-0.02	1.05	0.068
NSM	-0.93	-0.99	-0.50	0.009[c]	-0.91	-0.98	-0.38	0.016[c]	-0.97	-0.99	-0.79	0.001[c]	-0.97	-0.99	-0.80	<0.001[c]
DFI	0.10	0.05	0.16	<0.001[c]	0.10	0.04	0.16	<0.001[c]	0.05	-0.00	0.10	0.074	0.05	-0.00	0.11	0.057
NG	-0.34	-0.48	-0.16	0.001[c]	-0.31	-0.46	-0.13	0.002[c]	0.02	-0.17	0.25	0.848	0.01	-0.18	0.25	0.882
Zinc	-0.64	-0.70	-0.57	<0.001[c]	-0.64	-0.70	-0.56	<0.001[c]	-0.54	-0.62	-0.46	<0.001[c]	-0.54	-0.61	-0.45	<0.001[c]

[a] Confounding factors used in the adjusting model contained age, BMI, income, smoking status, and alcohol consumption.

[b] Effect estimates represented their percentage changes in association with a unit increase in FEI.

Abbreviations: TSM, total sperm motility; PSM, progressive sperm motility; NSM, normal sperm morphology; DFI, DNA fragmentation index; VCL, curvilinear velocity; VSL, straight line velocity; VAP, average path velocity; BCF, beat cross frequency; NG, Neutral glucosidase.

[c] $P<0.05$.

© 2022 Lv M et al. *JAMA Network Open.*

eTable 2. Associations of FEI With Semen Quality and Biochemistry Parameters of Seminal Plasma in Different Level of MDA Before and After Adjusting for Confounding Factors[a]

	Low level of MDA								High level of MDA							
	Crude				Adjusted				Crude				Adjusted			
	Effect estimate[b](%)	95%CI		*P* value	Effect estimate[b](%)	95%CI		*P* value	Effect estimate[b](%)	95%CI		*P* value	Effect estimate[b](%)	95%CI		*P* value
		lower	higher			Lower	higher			lower	higher			lower	higher	
TSM	-0.84	-0.99	2.39	0.235	-0.85	-0.99	1.97	0.205	-0.99	-0.99	-0.97	<0.001[c]	-0.99	-0.99	-0.97	<0.001[c]
PSM	-0.79	-0.98	1.74	0.228	-0.85	-0.98	1.00	0.149	-0.99	-0.99	-0.97	<0.001[c]	-0.99	-0.99	-0.97	<0.001[c]
VCL	-0.01	-0.05	0.02	0.354	-0.02	-0.05	0.01	0.247	-0.05	-0.08	-0.02	0.002[c]	-0.04	-0.08	-0.01	0.003[c]
VSL	-0.04	-0.08	0.00	0.078	-0.05	-0.09	0.00	0.061	-0.07	-0.11	-0.03	<0.001[c]	-0.07	-0.11	-0.03	<0.001[c]
VAP	-0.04	-0.08	0.00	0.073	-0.05	-0.09	0.00	0.053	-0.08	-0.11	-0.04	<0.001[c]	-0.07	-0.11	-0.03	<0.001[c]
BCF	0.25	-0.11	0.78	0.196	0.29	-0.08	0.83	0.145	0.61	0.15	1.26	0.006[c]	0.64	0.17	1.30	0.004[c]
NSM	-0.70	-0.95	0.92	0.198	-0.76	-0.96	0.64	0.144	-0.98	-0.99	-0.92	<0.001[c]	-0.97	-0.99	-0.91	<0.001[c]
DFI	0.02	-0.02	0.07	0.418	0.01	-0.03	0.06	0.553	0.11	0.07	0.15	<0.001[c]	0.10	0.06	0.15	<0.001[c]
NG	-0.01	-0.21	0.22	0.876	-0.00	-0.20	0.23	0.937	-0.32	-0.42	-0.19	<0.001[c]	-0.29	-0.41	-0.14	<0.001[c]
Zinc	-0.53	-0.61	-0.44	<0.001[c]	-0.51	-0.59	-0.42	<0.001[c]	-0.59	-0.64	-0.53	<0.001[c]	-0.59	-0.65	-0.52	<0.001[c]

[a] Confounding factors used in the adjusting model contained age, BMI, income, smoking status, and alcohol consumption.
[b] Effect estimates represented their percentage changes in association with a unit increase in FEI.
Abbreviations: TSM, total sperm motility; PSM, progressive sperm motility; NSM, normal sperm morphology; DFI, DNA fragmentation index; VCL, curvilinear velocity; VSL, straight line velocity; VAP, average path velocity; BCF, beat cross frequency; NG, Neutral glucosidase.
[c] $P<0.05$.

© 2022 Lv M et al. *JAMA Network Open.*

eTable 3. Associations of FEI With Semen Quality and Biochemistry Parameters of Seminal Plasma in Different Level of GSHPx Before and After Adjusting for Confounding Factors[a]

	Low level of GSHPx								High level of GSHPx							
	Crude				Adjusted				Crude				Adjusted			
	Effect estimate[b](%)	95%CI		*P* value	Effect estimate[b](%)	95%CI		*P* value	Effect estimate[b](%)	95%CI		*P* value	Effect estimate[b](%)	95%CI		*P* value
		lower	higher			lower	higher			Lower	higher			lower	higher	
TSM	-0.99	-0.99	-0.98	<0.001[c]	-0.06	-0.08	-0.03	<0.001[c]	-0.93	-0.99	0.41	0.083	-0.02	-0.04	0.00	0.088
PSM	-0.99	-0.99	-0.98	<0.001[c]	-0.06	-0.09	-0.04	<0.001[c]	-0.90	-0.99	0.35	0.082	-0.02	-0.04	0.00	0.073
VCL	-0.05	-0.09	-0.01	0.007[c]	-0.04	-0.08	-0.00	0.019[c]	-0.03	-0.07	0.00	0.055	-0.03	-0.06	0.00	0.083
VSL	-0.08	-0.13	-0.03	0.001[c]	-0.07	-0.12	-0.02	0.003[c]	-0.04	-0.09	0.00	0.051	-0.04	-0.09	0.00	0.052
VAP	-0.08	-0.13	-0.03	0.001[c]	-0.07	-0.12	-0.02	0.003[c]	-0.05	-0.09	0.00	0.065	-0.05	-0.09	0.00	0.136
BCF	0.33	-0.11	1.00	0.172	0.39	-0.07	1.08	0.109	0.36	-0.06	0.99	0.109	0.31	-0.10	0.93	0.160
NSM	-0.99	-0.99	-0.96	<0.001[c]	-0.99	-0.99	-0.95	<0.001[c]	-0.60	-0.94	2.11	0.376	-0.41	-0.92	3.81	0.615
DFI	0.16	0.10	0.21	<0.001[c]	0.14	0.09	0.20	<0.001[c]	0.02	-0.02	0.07	0.425	0.01	-0.03	0.06	0.565
NG	-0.36	-0.47	-0.22	<0.001[c]	-0.31	-0.44	-0.15	<0.001[c]	0.06	-0.15	0.34	0.582	0.03	-0.18	0.30	0.778
Zinc	-0.63	-0.68	-0.58	<0.001[c]	-0.62	-0.67	-0.56	<0.001[c]	-0.55	-0.62	-0.45	<0.001[c]	-0.54	-0.62	-0.45	<0.001[c]

[a] Confounding factors used in the adjusting model contained age, BMI, income, smoking status, and alcohol consumption.
[b] Effect estimates represented their percentage changes in association with a unit increase in FEI.
Abbreviations: TSM, total sperm motility; PSM, progressive sperm motility; NSM, normal sperm morphology; DFI, DNA fragmentation index; VCL, curvilinear velocity; VSL, straight line velocity; VAP, average path velocity; BCF, beat cross frequency; NG, Neutral glucosidase.
[c] $P<0.05$.

© 2022 Lv M et al. *JAMA Network Open.*

eTable 4. Effect Estimates and 95% CIs of Semen Quality Parameters With a 1-Unit Increase in FEI With and Without Adjustment for Smoking and Alcohol Consumption

	Age (year)							
	Model 1				Model 2			
	Effect estimate[a] (%)	95%CI		*P* value	Effect estimate[a] (%)	95%CI		*P* value
		lower	higher			lower	higher	
TSM	-0.99	-1.00	-0.98	<0.001 [b]	-0.99	-1.00	-0.99	<0.001 [b]
PSM	-0.99	-0.99	-0.98	<0.001 [b]	-0.99	-1.00	-0.98	<0.001 [b]
VCL	-0.05	-0.08	-0.02	<0.001 [b]	-0.06	-0.09	-0.03	<0.001 [b]
VSL	-0.08	-0.11	-0.04	<0.001 [b]	-0.09	-0.16	-0.04	<0.001 [b]
VAP	-0.08	-0.11	-0.04	<0.001 [b]	-0.09	-0.14	-0.05	<0.001 [b]
BCF	0.57	0.18	1.08	0.003 [b]	0.53	0.18	1.01	0.007 [b]
NSM	-0.98	-0.99	-0.93	<0.001 [b]	-0.98	-0.99	-0.93	<0.001 [b]
DFI	0.10	0.06	0.14	<0.001 [b]	0.12	0.07	0.12	<0.001 [b]
NG	-0.24	-0.35	-0.10	0.009 [b]	-0.33	-0.52	-0.12	0.015 [b]
Zinc	-0.61	-0.65	-0.56	<0.001 [b]	-0.53	-0.66	-0.50	<0.001 [b]

Model 1: This model was analyzed adjusting for confounding factors including age, BMI, income, smoking status, and alcohol consumption.
Model 2: This model was analyzed adjusting for confounding factors including age, BMI, income.
[a] Effect estimates represented their percentage changes in association with a unit increase in FEI.
Abbreviations: TSM, total sperm motility; PSM, progressive sperm motility; NSM, normal sperm morphology; DFI, DNA fragmentation index; VCL, curvilinear velocity; VSL, straight line velocity; VAP, average path velocity; BCF, beat cross frequency; NG, Neutral glucosidase.
[b] $P<0.05$.

© 2022 Lv M et al. *JAMA Network Open.*

eTable 5. Effect Estimates and 95% CIs of Semen Quality Parameters Associated With a 1-Unit Increase in FEI in Each Subgroup[a]

	Age (year)								BMI							
	≤30				>30				≤24 (underweight or normal)				>24 (overweight or obese)			
	Effect estimate[b](%)	95%CI		*P* value	Effect estimate[b](%)	95%CI		*P* value	Effect estimate[b](%)	95%CI		*P* value	Effect estimate[b](%)	95%CI		*P* value
		lower	higher			lower	higher			lower	higher			lower	higher	
TSM	-0.99	-1.00	-0.97	<0.001[c]	-0.99	-1.00	-0.96	<0.001[c]	-0.99	-1.00	-0.96	<0.001[c]	-0.99	-1.00	-0.98	<0.001[c]
PSM	-0.99	-0.99	-0.95	<0.001[c]	-0.99	-1.00	-0.93	<0.001[c]	-0.99	-1.00	-0.96	<0.001[c]	-0.99	-0.99	-0.95	<0.001[c]
VCL	-0.05	-0.08	-0.01	<0.001[c]	-0.05	-0.09	-0.00	0.032[c]	-0.05	-0.09	-0.01	<0.001[c]	-0.04	-0.08	-0.01	0.017[c]
VSL	-0.07	-0.11	-0.03	<0.001[c]	-0.07	-0.13	-0.01	0.014[c]	-0.09	-0.13	-0.04	<0.001[c]	-0.06	-0.10	-0.01	0.006[c]
VAP	-0.07	-0.11	-0.03	<0.001[c]	-0.08	-0.13	-0.02	0.013[c]	-0.09	-0.13	-0.04	<0.001[c]	-0.06	-0.11	-0.02	0.004[c]
BCF	0.52	0.09	1.13	0.007[c]	0.52	-0.06	1.48	0.090	1.05	0.36	2.08	<0.001[c]	0.20	-0.17	0.75	0.347
NSM	-0.96	-0.99	-0.80	<0.001[c]	-0.98	-0.99	-0.92	<0.001[c]	-0.97	-0.99	-0.85	<0.001[c]	-0.98	-0.99	-0.91	<0.001[c]
DFI	0.08	0.04	0.13	<0.001[c]	0.13	0.07	0.20	<0.001[c]	0.11	0.06	0.17	<0.001[c]	0.09	0.04	0.14	<0.001[c]
NG	-0.18	-0.32	-0.01	0.033[c]	-0.32	-0.49	-0.09	0.014[c]	-0.30	-0.44	-0.12	<0.012[c]	-0.17	-0.34	0.03	0.047[c]
Zinc	-0.58	-0.64	-0.51	<0.001[c]	-0.65	-0.71	-0.58	<0.001[c]	-0.61	-0.67	-0.53	<0.001[c]	-0.62	-0.68	-0.55	<0.001[c]

[a] This model was analyzed adjusting for confounding factors including age, BMI, income, smoking status, and alcohol consumption.
[b] Effect estimates represented their percentage changes in association with a unit increase in FEI.
Abbreviations: TSM, total sperm motility; PSM, progressive sperm motility; NSM, normal sperm morphology; DFI, DNA fragmentation index; VCL, curvilinear velocity; VSL, straight line velocity; VAP, average path velocity; BCF, beat cross frequency; NG, Neutral glucosidase.
[c] *P*<0.05.

© 2022 Lv M et al. *JAMA Network Open.*

eFigure 1. Flowchart of Participant Recruitment

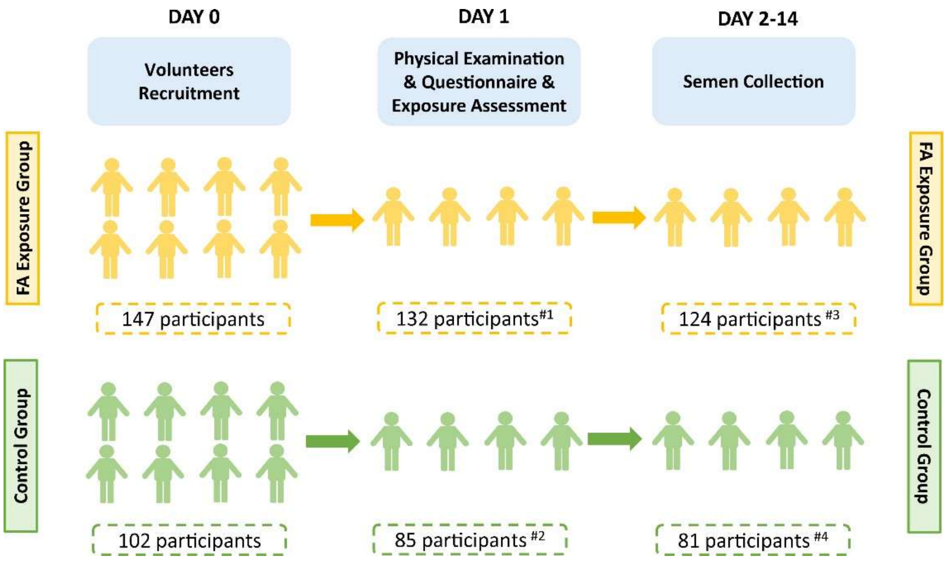

#1: 15 individuals were excluded, of which 4 participants lived in newly built or decorated rooms, 6 participants had genital abnormalities or other chronic diseases, and 5 participants did not complete the questionnaires.

#2: 17 individuals were excluded, of which 3 participants lived in newly built or decorated rooms, 5 participants had genital abnormalities or other chronic diseases, and 9 participants did not complete the questionnaires.

#3: 8 individuals were excluded since the incomplete collection of semen.

#4: 4 individuals were excluded since the incomplete collection of semen.

© 2022 Lv M et al. *JAMA Network Open.*

eFigure 2. Distribution of Sociodemographic Characteristics With and Without FA Exposure

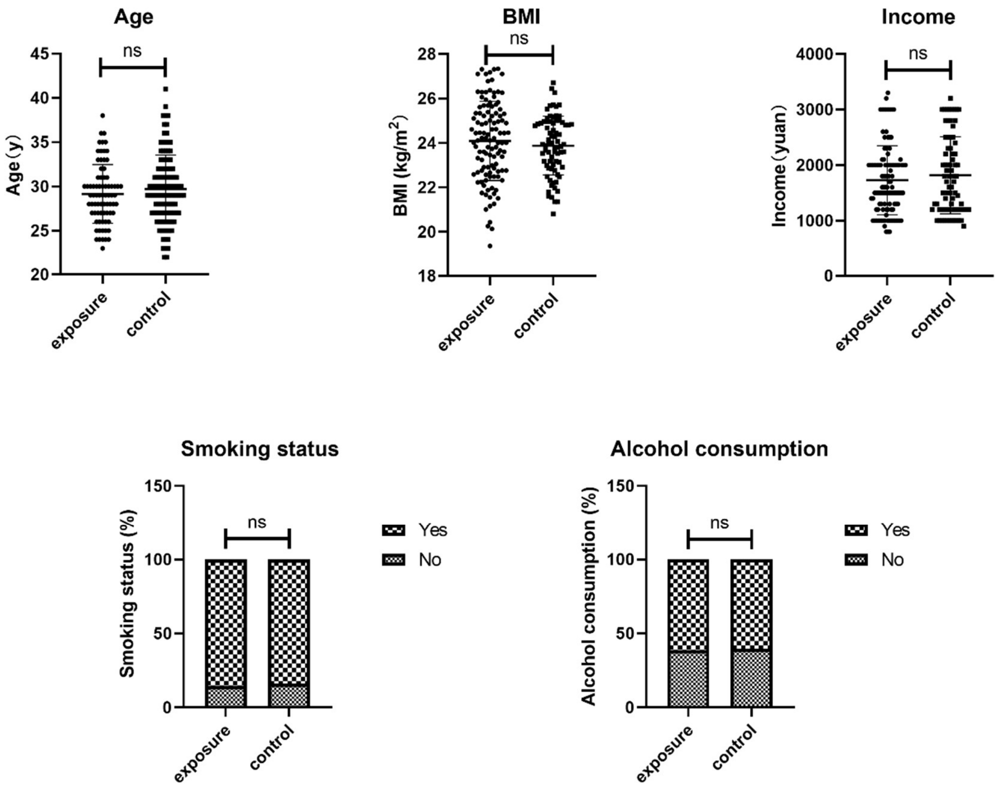

Abbreviations: BMI, body mass index.

ns: *P*>0.05.

© 2022 Lv M et al. *JAMA Network Open.*

eFigure 3. Percentage Changes in Semen Quality Associated With a 1-Unit Increase in FEI Before and After Adjusting for Confounding Factors

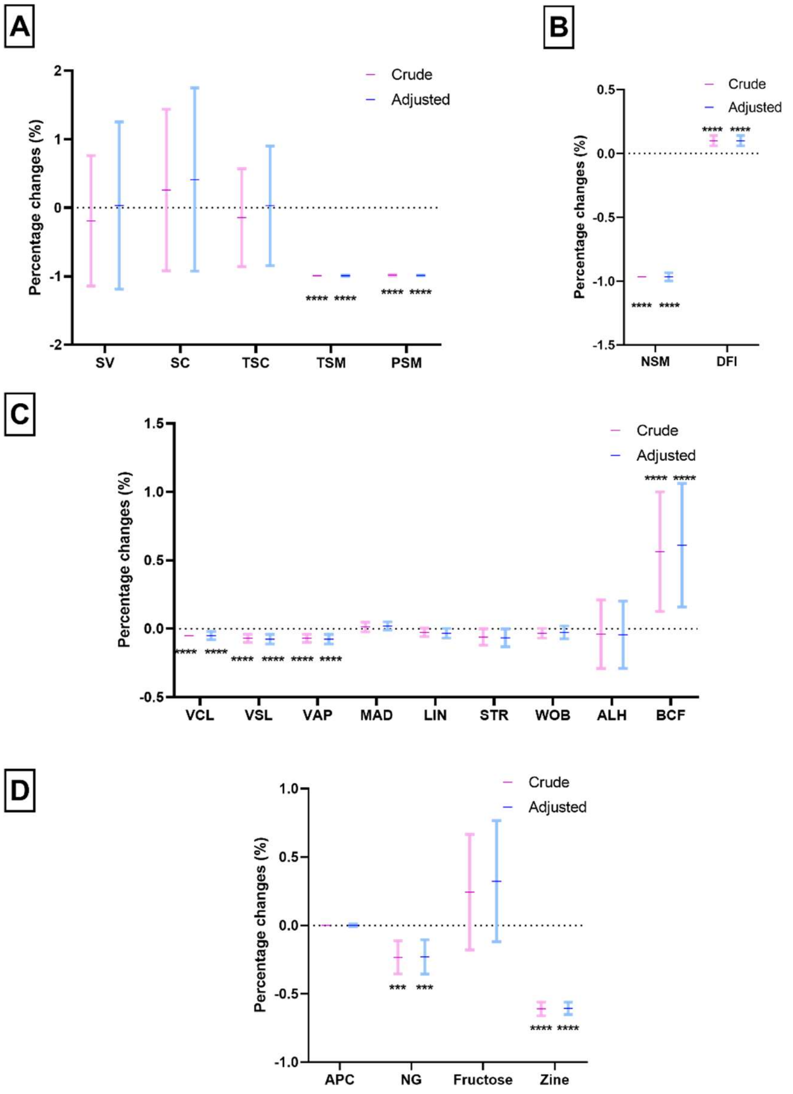

(A) conventional sperm parameters, (B) kinematic sperm parameters, (C) sperm morphology and DNA fragmentation index, (D) biochemistry parameters in seminal plasma.

Abbreviations: SV, semen volume; SC, sperm concentration; TSC, total sperm count; TSM, total sperm motility; PSM, progressive sperm motility; NSM, normal sperm morphology; DFI, DNA fragmentation index; VCL, curvilinear velocity; VSL, straight line velocity; LIN, linearity; VAP, average path velocity; STR, straightness; MAD, mean angular displacement; WOB, wobble; ALH, amplitude of lateral head displacement; BCF, beat cross frequency; APC, acid phosphatase; NG, Neutral glucosidase.

***: $P<0.005$, ****: $P<0.001$.

© 2022 Lv M et al. *JAMA Network Open.*

eFigure 4. Heatmap of the Associations Between FEI and Semen Quality in Different Groups of Antioxidant Defenses Before and After Adjusting for Confounding Factors

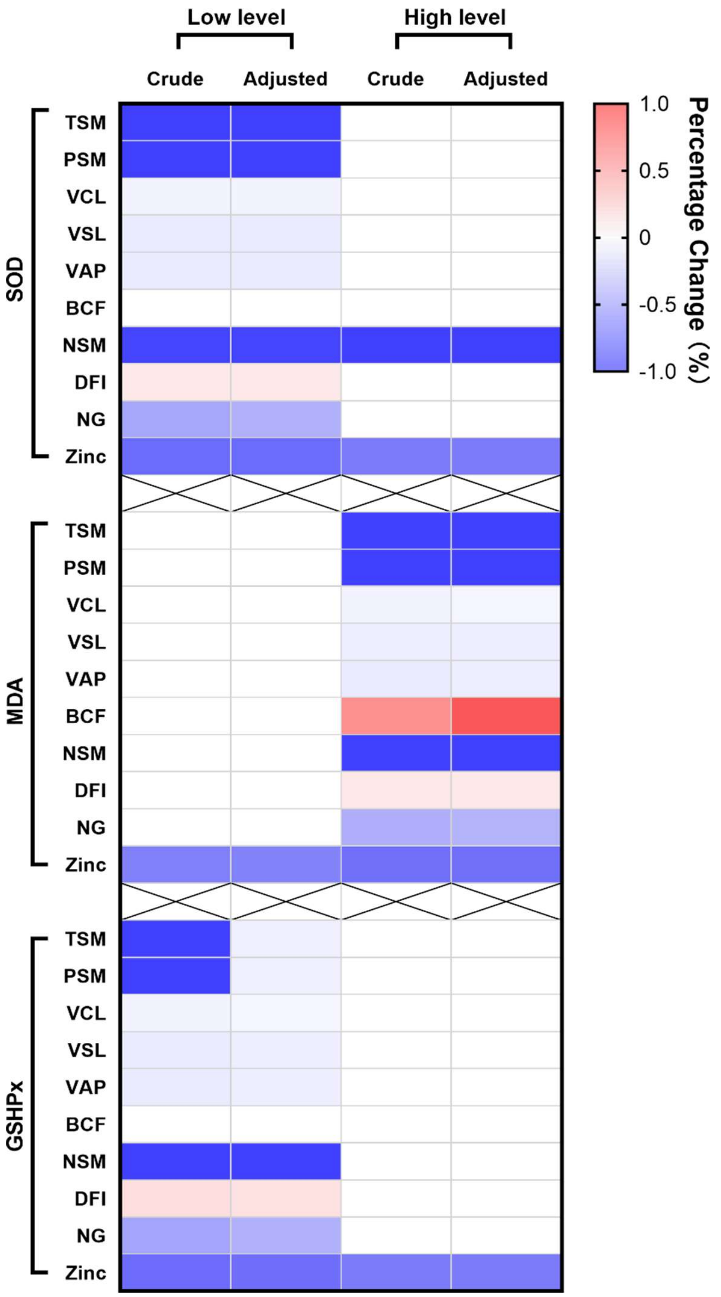

Blue indicated that a unit increase in FEI was associated with a significant decrease in this factor, pink indicated that a unit increase in FEI was associated with a significant increase in this factor, white indicated that a unit increase in FEI was not associated with this factor.

© 2022 Lv M et al. *JAMA Network Open.*

Abbreviations: TSM, total sperm motility; PSM, progressive sperm motility; NSM, normal sperm morphology; DFI, DNA fragmentation index; VCL, curvilinear velocity; VSL, straight line velocity; VAP, average path velocity; BCF, beat cross frequency; NG, Neutral glucosidase.

© 2022 Lv M et al. *JAMA Network Open.*

eFigure 5. Distribution of Semen Quality With and Without Occupational FA Exposure

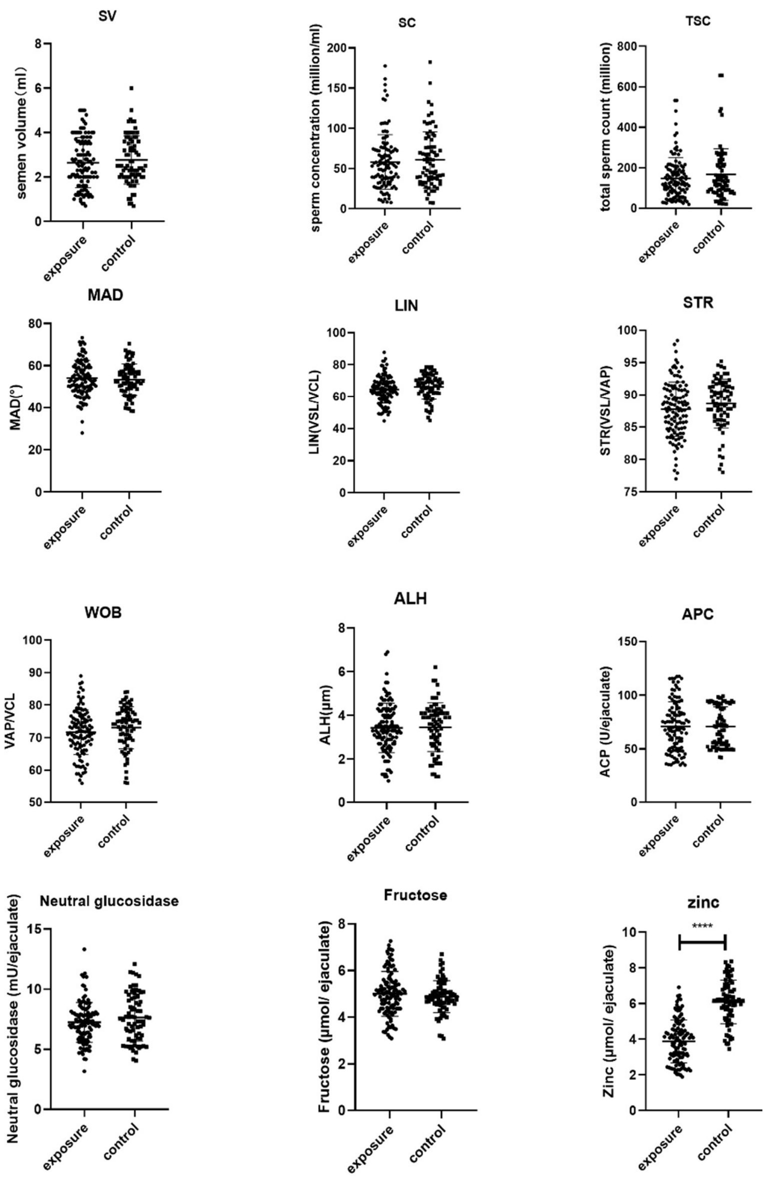

Abbreviations: SV, semen volume; SC, sperm concentration; TSC, total sperm count; LIN, linearity; STR, straightness; MAD, mean angular displacement; WOB, wobble; ALH, amplitude of lateral head displacement; ACP, acid phosphatase.

****: $P<0.001$.

© 2022 Lv M et al. *JAMA Network Open.*

eFigure 6. Significant Associations of FEI With Kinematic Sperm Parameters

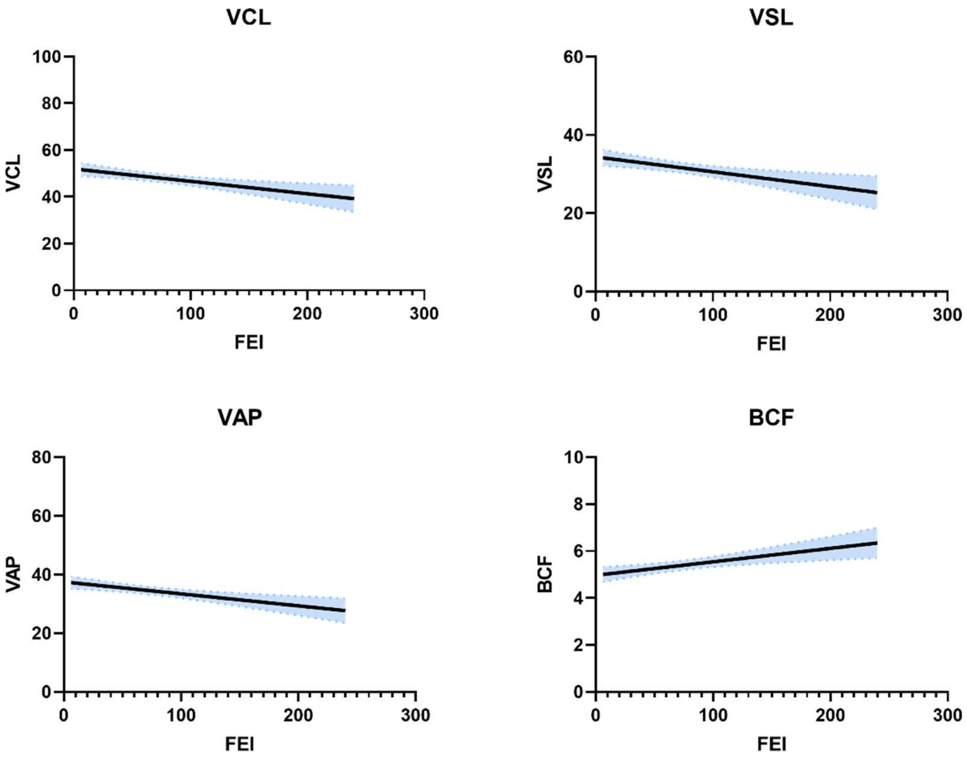

In all graphs, solid curves indicate beta and shaded areas indicate 95% CIs.

Abbreviations: VCL, curvilinear velocity; VSL, straight line velocity; VAP, average path velocity; BCF, beat cross frequency.

© 2022 Lv M et al. *JAMA Network Open.*
